# Heparin-Binding Protein and Transplant-Associated Inflammation: Emerging Roles in Infection, Ischemia–Reperfusion Injury, and Allograft Dysfunction

**DOI:** 10.3390/jcm15145365

**Published:** 2026-07-09

**Authors:** Chengchang Zhang, Ruozhu Li, Chen Dai

**Affiliations:** 1 Institute of Organ Transplantation, Tongji Hospital, Tongji Medical College, Huazhong University of Science and Technology, Wuhan 430074, China; u202110370@hust.edu.cn (C.Z.); u202416564@hust.edu.cn (R.L.); 2 Key Laboratory of Organ Transplantation, Ministry of Education, Tongji Hospital, Tongji Medical College, Huazhong University of Science and Technology, Wuhan 430074, China; 3 NHC Key Laboratory of Organ Transplantation, Tongji Hospital, Tongji Medical College, Huazhong University of Science and Technology, Wuhan 430074, China; 4 Key Laboratory of Organ Transplantation, Chinese Academy of Medical Sciences, Tongji Hospital, Tongji Medical College, Huazhong University of Science and Technology, Wuhan 430074, China; 5 Hubei Provincial Clinical Research Center for Organ Transplantation, Tongji Hospital, Tongji Medical College, Huazhong University of Science and Technology, Wuhan 430074, China; 6 Tongji Hospital, Tongji Medical College, Huazhong University of Science and Technology, Wuhan 430074, China

**Keywords:** heparin-binding protein (HBP), organ transplantation, biomarker, inflammatory pathway, acute rejection (AR)

## Abstract

Heparin-binding protein (HBP) is an activation-dependent neutrophil granule protein involved in innate immune activation, endothelial barrier disruption, and inflammatory tissue injury. Although HBP has been extensively investigated in infectious diseases, particularly sepsis, its potential relevance to transplantation has only recently attracted attention. Several post-transplant complications, including infection, ischemia–reperfusion injury (IRI), microvascular dysfunction, and allograft rejection, share common pathological features such as neutrophil activation, endothelial injury, and excessive inflammatory amplification, suggesting a possible mechanistic role for HBP. This review summarizes the current understanding of HBP biology and evaluates its potential contribution to transplant-associated complications. We also discuss the feasibility of using HBP as an early biomarker for infection surveillance, inflammatory risk stratification, and graft injury monitoring. Furthermore, emerging therapeutic approaches targeting HBP or HBP-mediated vascular inflammation, including neutralizing antibodies, heparin derivatives, and albumin, are critically assessed. While experimental studies have provided preliminary evidence supporting HBP-targeted intervention, clinical validation in transplant populations remains insufficient. Future studies should define the temporal dynamics, cellular sources, and context-specific effects of HBP after transplantation. Collectively, available evidence indicates that HBP may serve as a potential biomarker and therapeutic target, but its clinical application requires further validation through mechanistic and prospective studies.

## 1. Introduction

In recent decades, with the development of transplantation technology, transplantation has gradually become one of the crucial treatments for a variety of end-stage diseases [1,2]. However, organ transplantation is often accompanied by significant immune rejection, so long-term immunosuppressive drugs are required to maintain stable graft function. But immunosuppressive therapy also significantly increases the risk of infection, especially in the early stages of transplantation [3]. Therefore, accurate and timely infection monitoring is of great significance for the prognosis of transplant patients. Currently, commonly used infection biomarkers include procalcitonin (PCT) and C-reactive protein (CRP) [4]. However, the elevation of these markers usually lags behind the onset of symptoms of infection, making it difficult to meet the need for early warning of infection after transplantation [5]. Therefore, exploring more sensitive and specific infection markers has become the focus of current research. In recent years, studies in specific non-transplant clinical settings have reported that HBP may have diagnostic or prognostic value, particularly in sepsis-induced acute kidney injury and in the diagnosis of bacteremia among patients with sepsis, where HBP showed relatively high diagnostic performance compared with PCT and CRP [6,7]. Based on this, HBP is expected to be used as a potential biomarker for early identification and monitoring of infection after organ transplantation.

Neutrophil-derived HBP, also known as CAP37, is a member of the serine protease family, which is mainly stored in neutrophil azurophilic granules and released into the bloodstream during infection. HBP can chemotaxis and activate immune cells, while increasing vascular endothelial permeability and promoting inflammatory cell recruitment. However, in severe infections, its excessive release can lead to vascular leakage, immune imbalance, and organ damage [8]. Clinically, HBP has become an important marker for the early diagnosis and prognosis assessment of sepsis. It has a high specificity and a short half-life in bacterial infections, which can dynamically reflect the disease and treatment response and help evaluate the effect of anti-infective therapy [9,10,11,12].

Nevertheless, research on HBP in organ transplantation remains extremely limited, and its role and application value in post-transplant infection, rejection, and ischemia–reperfusion injury (IRI) have not yet been fully established. Rather than presenting HBP as an established clinical biomarker or therapeutic target in transplantation, this narrative and biomarker-focused review summarizes the current evidence, identifies areas in which HBP has been directly studied in transplant recipients, and distinguishes these data from mechanistic hypotheses extrapolated from sepsis, lung injury, burns, cardiothoracic surgery, and other non-transplant inflammatory models. In particular, the possible roles of HBP in post-transplant infection, rejection, IRI, chronic rejection, and graft vasculopathy are discussed cautiously as potential mechanisms that require further validation. More prospective transplant-specific cohorts and organ-specific mechanistic studies are needed before the clinical utility of HBP in transplantation can be established.

## 2. Basic Biological Characteristics of HBP

HBP is an approximately 37 kDa basic granulocyte protein belonging to the serine protease family, which has structural similarity but no enzymatic activity. It is mainly stored in the azurophilic granules and secretory vesicles of neutrophils, and exists in small amounts on the surface of cell membranes. HBP can be quickly released when the body is strongly stimulated. Certain stimuli (e.g., N-Formyl-Met-Leu-Phe, fMLP) can also selectively induce secretory vesicle release [13]. In addition to neutrophils, monocytes, macrophages, and vascular endothelial cells can also express HBP in small amounts, but neutrophils are still its main source [14].

**Figure 1:**
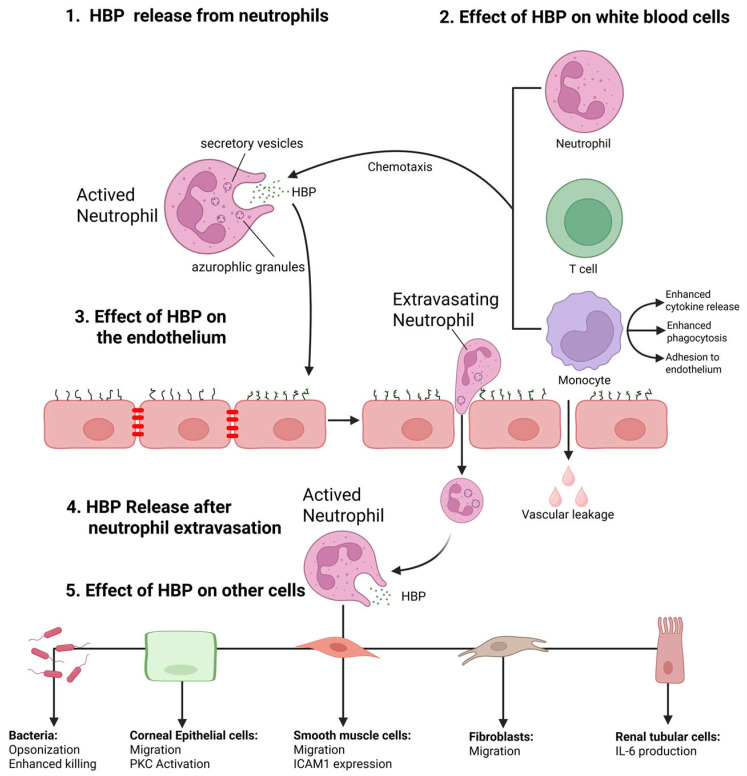
Biological release and functional effects of heparin-binding protein (HBP). HBP is mainly stored in neutrophil azurophilic granules and secretory vesicles and is released upon neutrophil activation. Extracellular HBP promotes neutrophil chemotaxis, leukocyte activation, cytokine release, phagocytosis, and adhesion to the endothelium. HBP also increases endothelial permeability, facilitates neutrophil extravasation, and may contribute to vascular leakage and inflammatory amplification. After extravasation, activated neutrophils can further release HBP within tissues. In addition to leukocytes and endothelial cells, HBP may affect other cell types, including bacteria, epithelial cells, smooth muscle cells, fibroblasts, and renal tubular cells.

HBP has both antibacterial and inflammatory regulatory functions. On one hand, it binds to and neutralizes Gram-negative lipopolysaccharides (LPS) and is directly involved in innate immune defense [15]. On the other hand, as an inflammatory “alarm hormone”, HBP enhances the immune response by inducing monocyte migration and the release of pro-inflammatory factors (such as IL-6, TNF-α, IL-8), and increases vascular permeability to promote the recruitment of inflammatory cells such as T cells. In addition, HBP can also protect endothelial cells from apoptosis and reduce tissue damage under oxidative stress. In summary, HBP plays a central role in the anti-infective and inflammatory cascade, laying a theoretical foundation for its potential role as a biomarker and an intervention target in organ transplantation (Figure 1).

## 3. The Mechanism of HBP-Mediated Inflammatory Response and Its Potential Role in Organ Transplantation

### 3.1. HBP Enhanced-Vascular Permeability and Its Pathological Significance

During infection, HBP activates multiple signaling pathways by binding to endothelial cell surface glycosaminoglycans, leading to cell contraction and connexin degradation, thereby disrupting the vascular barrier and increasing permeability. Relevant studies suggest that its role involves key signaling axes such as PKC/Rho kinase, β2 integrin-PI3K and TRIM21–P65, which ultimately regulate the barrier function and metabolic state of endothelial cells, revealing the core mechanisms of HBP in vascular permeability and inflammatory damage. HBP enhances the stability of TRIM21 by inhibiting K48 ubiquitination. TRIM21 binds to P65 and promotes its K63-linked ubiquitination, thereby facilitating its nuclear translocation, which in turn regulates the permeability and glycolysis of HPMECs [16,17,18]. HBP can bind to transforming growth factor-β receptor type 2 (TGF-β-R2) as a ligand. Glutathione S-transferase pull-down analysis showed that HBP mainly interacted with the extracellular domain of TGF-β-R2. HBP induces acute lung injury and vascular leakage by activating the TGF-β/SMAD2/3 signaling pathway [19].

Vitro studies have shown that HBP can induce calcium-dependent cytoskeletal rearrangement, leading to the formation of endothelial intercellular spaces and disrupting barrier function. In vivo, it significantly enhances microvascular permeability and promotes macromolecular extravasation, resulting in vascular leakage, tissue edema and organ dysfunction, which manifests as capillary leakage syndrome in cardiopulmonary shunt surgery [20,21]. In addition, HBP can further exacerbate endothelial damage by displacing endothelial surface kininogen and activating the kinin-releasing enzyme-kinin system (KKS) to produce bradykinin and act on B2 receptors. Relevant studies suggest that this pathway amplifies the inflammatory response in diseases such as vasculitis in children, so KKS is considered one of the potential targets for inflammatory intervention [22].

In sepsis, HBP induces acute lung injury by enhancing endothelial permeability and activating glycolytic pathways; In renal sepsis, HBP accelerates the onset and progression of acute kidney injury (AKI) by promoting vascular leakage [18,20,23,24]. Neutrophil-derived HBP and myeloperoxidase (MPO) play an important role in triggering vascular leakage in the early stages of severe burns. After severe burns, activated neutrophils rapidly increase and secrete HBP and MPO. Elevated HBP triggers vascular leakage under the synergistic effect of MPO, leading to generalized edema and burn shock. In addition, the catalytic product of MPO, Hypochlorous acid, rather than MPO itself, triggers the shedding of the CD44 extracellular domain in vascular endothelial cells, thereby damaging the glycocalyx structure. Glycocalyx damage causes strong adhesion of neutrophils and increased vascular leakage [25]. In the burn shock stage, neutrophil function was significantly reduced, chemotaxis and phagocytosis decreased, and bactericidal function was abnormal. In the burn shock stage, increased release of HBP and increased expression of P2RX1 on the surface of neutrophils were associated with fluid leakage and decreased chemotaxis, respectively. The combination of HBP concentration and neutrophil P2RX1 expression in plasma can better predict neutrophil dysfunction in burn patients. Targeted restoration of neutrophil function may be a feasible therapeutic intervention that can help reduce fluid loss during shock and the severity of subsequent infection [26].

Although most of the above mechanisms have been characterized in sepsis, acute lung injury, burns, cardiopulmonary bypass, and other non-transplant inflammatory settings, they may be biologically relevant to transplantation. During organ procurement, preservation, reperfusion, and early post-transplant inflammation, the graft endothelium is exposed to ischemia–reperfusion injury, complement activation, neutrophil infiltration, cytokine release, and microvascular stress. These processes share several pathological features with the inflammatory models described above, including endothelial barrier disruption, vascular leakage, leukocyte adhesion, glycocalyx injury, and metabolic activation of endothelial cells. Therefore, HBP-mediated regulation of endothelial permeability may provide a mechanistic basis for studying post-transplant complications such as graft edema, microvascular dysfunction, ischemia–reperfusion injury, acute rejection-associated endothelial injury, and early allograft dysfunction.

Together, these studies suggest a potential pathogenic role of HBP in infection-related and sterile inflammatory responses involving endothelial barrier dysfunction. In the context of transplantation, these findings provide a rationale for further investigation of HBP as a candidate biomarker and mechanistic mediator of transplant-associated endothelial injury, while its clinical value as a diagnostic marker or therapeutic target requires validation in transplant-specific studies.

### 3.2. HBP-Mediated Endothelial Permeability and Leukocyte Recruitment

In addition to enhancing vascular permeability, HBP may amplify inflammatory responses by promoting leukocyte recruitment. HBP has been reported to interact with CC chemokine receptor 2 (CCR2) and formyl peptide receptor-like 1 (FPRL1) on monocytes, thereby inducing monocyte migration toward sites of infection and inflammation. In endothelial cells, HBP can also promote the secretion of chemokines such as monocyte chemoattractant protein-1 (MCP-1) through activation of the FAK/PI3K/AKT and p38 MAPK/NF-κB pathways, further enhancing inflammatory cell recruitment [16].

**Figure 2:**
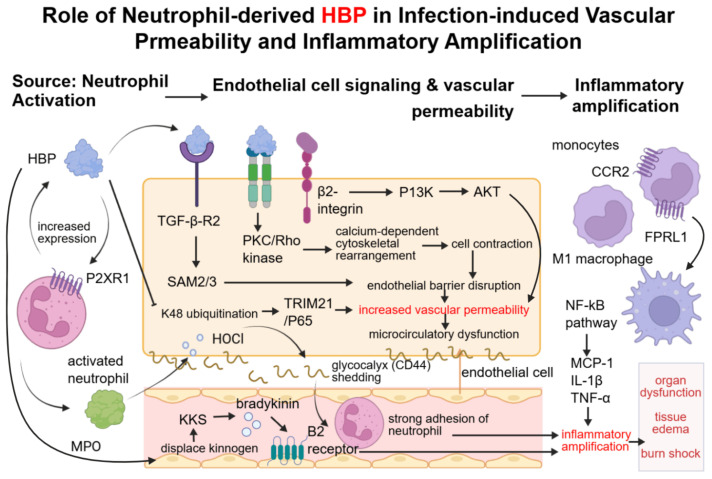
Role of Neutrophil-derived HBP in Infection-induced Vascular Permeability and Inflammatory Amplification.

HBP may also promote the release of inflammatory mediators, including interleukin-1β (IL-1β) and tumor necrosis factor-α (TNF-α), thereby indirectly facilitating the accumulation of immune cells at inflammatory sites. This amplification effect may contribute to host defense during infection; however, excessive or sustained HBP activation may aggravate endothelial injury, vascular leakage, uncontrolled inflammation, and organ damage [6]. These mechanisms, although mainly characterized in infection and other non-transplant inflammatory models, may provide a biological basis for further investigation of HBP in transplant-associated endothelial dysfunction and inflammatory injury (Figure 2).

### 3.3. HBP-Mediated Macrophage Activation and Non-Classical Inflammatory Amplification

Beyond its effects on endothelial permeability and leukocyte recruitment, HBP may also regulate inflammatory responses through macrophage activation and other non-classical mechanisms. HBP has been reported to promote M1 macrophage activation, lactate accumulation, and the expression of pro-inflammatory mediators, including IL-1β, inducible nitric oxide synthase (iNOS), TNF-α, and interleukin-6 (IL-6), partly through the NF-κB pathway, with lactate acting as an important regulatory mediator [27]. In addition, HBP may induce macrophage secretion of TNF-α and interferon-γ (IFN-γ) and enhance phagocytosis by upregulating Fcγ receptors [28].

HBP release may also interact with other inflammatory mediators in local host defense. For example, intrabronchial exposure to lipopolysaccharide (LPS), a Toll-like receptor 4 (TLR4) agonist, can stimulate the concomitant release of HBP and IL-26 in the human airway. IL-26 may act as a co-stimulatory factor for HBP release from neutrophils, suggesting a potential synergistic role of HBP and IL-26 in mucosal immune defense [29].

In addition to its immunomodulatory effects, HBP has been reported to exert direct antibacterial and tissue-repair-related activities. Kasus-Jacobi et al. found that topical application of HBP promoted corneal epithelial regeneration in mice, and HBP-derived peptides reproduced the antibacterial and wound-healing effects of the full-length protein, possibly through mechanisms related to TLR4 inhibition [30].

Taken together, HBP may contribute to inflammatory amplification through multiple mechanisms, including macrophage activation, metabolic regulation, cytokine induction, interaction with IL-26, and direct antibacterial activity. However, most of these findings are derived from non-transplant inflammatory models. Therefore, whether these mechanisms are involved in post-transplant infection, rejection, ischemia–reperfusion injury, or graft dysfunction remains to be determined in transplant-specific studies (Table 1).

## 4. The Clinical Potential and Application Prospects of HBP in Organ Transplantation

### 4.1. Mechanism of Action of HBP in Organ Transplantation-Related Pathological Processes

Organ transplantation surgery and perioperative management often trigger a strong aseptic inflammatory response. Factors such as surgical manipulation, IRI, and the possible use of extracorporeal bypass activate the receptor’s innate immune system, causing the release of large inflammatory mediators. Among them, neutrophil recruitment and degranulation are important links. HBP is released in large quantities at this stage. In the context of organ transplantation, HBP may be deeply involved in a variety of intraoperative and postoperative pathophysiological processes through its pro-inflammatory and immunomodulatory effects, which has an important impact on transplant outcomes. Its potential involvement mechanisms include intraoperative/postoperative systemic inflammatory responses, primary graft dysfunction or delayed recovery of graft function (PGD/DGF), and immune-mediated AR [31]. The above effects suggest that HBP is not only a core mediator of aseptic inflammatory amplification, but may also constitute an early intervention target and risk predictor for transplant-related complications [32,33,34]. However, it should be emphasized that direct evidence for marked HBP release during transplant-associated inflammatory responses remains limited. Current evidence is mainly derived from lung transplantation studies and from non-transplant surgical or inflammatory models, such as cardiopulmonary bypass, sepsis, and acute lung injury. Therefore, the potential involvement of HBP in intraoperative and postoperative inflammation after organ transplantation should be interpreted cautiously.

Lung transplantation studies have shown that neutrophil-derived HBP is significantly elevated in the early postoperative period. If cardiopulmonary bypass (CPB) is used intraoperatively, it can induce a stronger neutrophil activation and degranulation response, further promoting the release of neutrophil mediators such as HBP and MPO. The explosive release of these inflammatory mediators in the perioperative period not only constitutes a key driver of early graft injury but may also lead to systemic inflammatory response syndrome (SIRS), which adversely affects the systemic stability and recovery process of patients after surgery. Therefore, HBP is considered to be one of the important inflammatory mediators of IRI and postoperative inflammatory response during organ transplantation. These findings suggest that overactivation of the innate immune system should be closely monitored and appropriately regulated in the perioperative period to reduce the risk of postoperative complications and improve transplant outcomes [35,36].

Acute graft rejection (AR) has traditionally been thought to be mainly mediated by the adaptive immune system (such as T cells, B cells, etc.), but in recent years, the synergistic role of the innate immune system in it has gradually attracted attention. Inflammatory monocytes are the main components of cellular infiltration in human AR kidney grafts. Immunomodulatory nanoparticles (IMPs) can bind to circulating inflammatory monocytes through specific scavenger receptor MARCO, causing them to turn to the spleen and subsequently undergo apoptosis [37]. HBP, as a product of neutrophil activation and degranulation, may play a supporting pathogenic role in rejection. In antibody-mediated rejection (ABMR), MPO is produced by receptor-derived myeloid cells and is significantly expressed during AR, which can further promote graft tissue damage by regulating the activation status of natural killer (NK) cells and the function of monocytes/macrophages [38]. Although this evidence does not directly establish HBP as a mediator of ABMR, it supports the broader concept that neutrophil- and myeloid-cell-associated inflammatory pathways may interact with adaptive immune responses during rejection. Based on these mechanisms, HBP may participate in AR and ABMR-related endothelial injury by increasing microvascular permeability and facilitating inflammatory cell infiltration. Measurement of HBP in serum, urine, bronchoalveolar lavage fluid, graft tissue, or local graft drainage may help clarify whether HBP is associated with rejection activity or rejection subtype. However, the role of HBP in AR and ABMR remains largely hypothesis-generating. Further transplant-specific studies are needed to determine whether HBP can serve as a useful biomarker or therapeutic target in acute rejection.

For chronic rejection, there is no direct evidence that HBP plays a clear role in its occurrence and development. However, given HBP’s critical role in acute inflammation and endothelial dysfunction, its potential involvement in chronic rejection warrants further investigation. Chronic rejection is usually characterized by persistent damage to the vascular endothelium, vascular smooth muscle cell proliferation, and interstitial fibrosis, which is mostly driven by continuous immune stimulation, such as low levels of donor receptor antibodies and chronic microinflammatory state. In terms of clinical manifestations, chronic rejection often presents with progressive graft hypofunction, accompanied by non-specific manifestations such as proteinuria and hypertension. Its histopathological changes are usually insidious and lack typical inflammatory cell infiltration, but the multilayered and fibrotic features of the microvasculature of transplanted organs are highly specific. Taking kidney transplantation as an example, the most effective prevention strategies currently focus on controlling chronic damage caused by immune and non-immune factors, and using non-nephrotoxic immunosuppressive regimens for long-term maintenance therapy [39,40]. Although no studies have directly demonstrated the pathogenic role of HBP in chronic rejection, HBP-mediated mechanisms may provide a biologically plausible link to chronic endothelial dysfunction, ultimately contributing to chronic endothelial dysfunction and graft structural remodeling [18,22,41].

Chronic graft vasculopathy limits graft and recipient survival after heart transplantation, and it can occur in other vascularized organ transplants as well. Cardiac allograft vasculopathy (CAV) is a pathological, immune-mediated remodeling of graft vessels that impairs perfusion and is the leading cause of advanced graft loss. Some features of CAV may be common to other immune-mediated vascular diseases. Both cellular and antibody-mediated immune responses against the vascular endothelium can trigger maladaptive fibrocyte damage in the arteries, and it can be speculated that HBP is also involved. Currently, the CAV biomarkers used in clinical practice include CRP, so HBP may be explored as a candidate biomarker, but this possibility requires clinical validation [42,43].

It is worth noting that chronic rejection often involves the combined action of multiple mechanisms, including low levels of persistent antibodies, endothelial-immune interaction imbalance, and abnormal tissue repair processes, and HBP, as a key mediator connecting neutrophils, endothelial cells and inflammatory signals, may represent one of several inflammatory mediators linking neutrophil activation, endothelial dysfunction, and tissue remodeling. In the future, it is necessary to further clarify the expression dynamics, cell origin and pathological mechanism of HBP in chronic rejection through long-term follow-up cohort studies, in situ detection at the tissue level and functional experimental models, so as to explore its potential value in early identification and intervention of chronic rejection.

In summary, HBP may be mechanistically relevant to several organ transplantation-related pathological processes through its chemotactic, pro-inflammatory, and endothelial barrier-disruptive functions. These properties provide a biological basis for exploring its potential involvement in acute rejection, ABMR-associated endothelial injury, chronic rejection, and graft vasculopathy. Future studies should evaluate the expression dynamics and cellular sources of HBP in different transplant contexts, determine whether HBP levels are associated with acute or chronic rejection in large prospective cohorts, and clarify whether HBP-related pathways contribute to microvascular injury and graft remodeling. In addition, experimental transplantation models are needed to assess whether targeting HBP or its downstream inflammatory pathways could provide therapeutic benefit, either alone or in combination with existing immunosuppressive strategies.

### 4.2. Analysis of the Possible Role of HBP in IRI

IRI is a major determinant of early graft injury after transplantation and is characterized by oxidative stress, mitochondrial dysfunction, endothelial activation, and inflammatory amplification [44,45,46]. During ischemia, ATP depletion, intracellular acidosis, calcium overload, and endothelial stress promote the release of damage-associated molecular patterns (DAMPs), including high-mobility group box 1 (HMGB1), ATP, and DNA fragments. After reperfusion, restored oxygen supply further enhances mitochondrial reactive oxygen species (ROS) production, aggravates mitochondrial damage, and activates innate immune responses [47,48,49]. Neutrophil recruitment and activation are important components of the effector phase of IRI. In hepatic IRI, DAMPs activate Kupffer cells through pattern-recognition receptors, leading to the release of pro-inflammatory mediators such as TNF-α and IL-1β. These signals further recruit neutrophils, which can damage hepatocytes and sinusoidal endothelial cells through the release of proteases, myeloperoxidase (MPO), ROS, and other inflammatory mediators. Endothelial activation also promotes leukocyte adhesion, platelet interaction, sinusoidal obstruction, and the “no-reflow” phenomenon, thereby aggravating microcirculatory dysfunction and graft injury [48,49,50].

Within this inflammatory framework, HBP may represent a biologically plausible neutrophil-derived mediator linking neutrophil activation to endothelial barrier disruption and inflammatory amplification in IRI. Activated neutrophils can release HBP during inflammatory responses, and studies in inflammatory models have shown that HBP can promote leukocyte recruitment, increase endothelial permeability, activate monocytes/macrophages, and enhance NF-κB-related inflammatory signaling. These biological properties are consistent with several key pathological features of IRI, including vascular leakage, tissue edema, microcirculatory disturbance, and sustained inflammatory injury. Therefore, HBP may contribute to IRI-associated graft damage, particularly in settings where neutrophil degranulation and endothelial dysfunction are prominent. However, this proposed role stll require validation in transplant-specific IRI studies.

Clinically, IRI can directly damage the donor graft and is the main cause of PGD/DGF, affecting the short-term survival of patients. Myocardial ischemia–reperfusion injury (MIRI) and intramyocardial hemorrhage (IMH) can be accompanied by systolic dysfunction and poor cardiac remodeling [51,52]. Potential intervention strategies for IRI include Machine Perfusion (MP), Ischemic Preconditioning (IPC), and pharmacological interventions. MP, especially hypogenic mechanical perfusion (HMP), normothermic mechanical perfusion (NMP), and hypogenic oxygenation perfusion (HOPE), is considered a major breakthrough in the field of transplantation in recent years. They not only reduce IRI, but can also be used to evaluate and repair organs for transplantation [48,53,54]. IPC requires the application of a short ischemia–reperfusion cycle before prolonged ischemia to stimulate endogenous protective mechanisms in the liver [55]. Although these interventions are not specifically directed at HBP, they provide clinically relevant settings in which HBP-associated neutrophil and endothelial responses could be further evaluated.

Taken together, HBP may serve as a mechanistically relevant link between neutrophil activation, endothelial dysfunction, microcirculatory disturbance, and inflammatory amplification in IRI. Future studies should investigate the temporal changes in HBP in transplant recipients, particularly in liver, kidney, lung, and heart transplantation, and evaluate whether HBP levels are associated with IRI severity, PGD/DGF, or early graft dysfunction. Experimental transplantation models may further clarify whether targeting HBP or HBP-related inflammatory pathways can attenuate IRI-associated graft injury.

### 4.3. HBP as a Biomarker for Organ Transplant-Related Infections

As mentioned earlier, HBP can be rapidly released during the early phase of infection and has been investigated as a sensitive biomarker of neutrophil activation and inflammatory injury [24]. In the field of organ transplantation, the most direct evidence currently comes from lung transplantation. Stjärne Aspelund et al. measured HBP in bronchoalveolar lavage fluid (BALF) from lung transplant recipients and showed that BALF HBP was a sensitive marker for detecting pulmonary infection, with diagnostic performance superior to lysozyme and several inflammatory cytokines [36]. This finding is particularly relevant to lung transplantation because infectious complications and non-infectious events, such as acute rejection (AR), may show overlapping clinical and radiological features, while conventional microbial culture can be time-consuming and may have limited sensitivity. Therefore, BALF HBP may serve as a useful local candidate biomarker for pulmonary infection surveillance after lung transplantation and may assist in differentiating infectious from non-infectious post-transplant complications.

Outside the transplant setting, HBP has been evaluated as a biomarker in several infectious and inflammatory diseases, most notably sepsis. Studies have reported its potential value in neonatal sepsis, systemic infection, meningitis, hospital-acquired meningitis and ventriculitis, postoperative myocardial injury-related cardiogenic shock (MIRCS), and adult-onset Still’s disease (AOSD) [7,11,56,57,58,59,60,61,62,63]. In these settings, elevated HBP levels have been associated with early neutrophil activation, infection status, inflammatory severity, organ dysfunction, or adverse outcomes, and in some studies HBP showed diagnostic performance comparable or superior to conventional markers such as PCT, CRP, lactate, or cerebrospinal fluid lactate. These non-transplant findings support the broader concept that HBP may serve as an early indicator of neutrophil-driven inflammation and infection-related organ injury, providing a rationale for further evaluation of HBP in transplant recipients.

The relevance of these sepsis and infection studies to transplantation lies in the clinical features of transplant recipients. After transplantation, patients often receive immunosuppressive therapy, which may blunt fever, leukocytosis, and other typical signs of infection. In addition, postoperative inflammation, ischemia–reperfusion injury, rejection, and infection may coexist or present with overlapping manifestations. A biomarker that reflects early neutrophil activation could therefore be valuable as part of a multi-parameter strategy for post-transplant infection surveillance and inflammatory risk stratification. In sepsis-related lung injury, neutrophil activation products such as HBP and MPO increase early during disease progression and may remain detectable for longer than some pro-inflammatory cytokines, such as IL-6 and IL-8 [64,65]. These characteristics may be useful in transplant settings where early diagnosis and timely intervention are essential.

HBP has also been included in biomarker-based studies of sepsis-associated acute kidney injury. Wiersema, Jukarainen, and colleagues identified sepsis-related acute kidney injury subphenotypes with different prognostic features using multiple biomarkers, including HBP [66]. Although this evidence is not derived from transplant recipients, it may be relevant to kidney transplantation because DGF, infection, and perioperative inflammation are closely linked to renal microvascular injury and innate immune activation. Future studies could evaluate whether HBP helps define inflammatory phenotypes or risk categories after kidney transplantation.

Another clinically relevant issue is the interpretation of HBP levels during renal replacement therapy. HBP has a molecular weight of approximately 37 kDa, which may influence its clearance during continuous renal replacement therapy (CRRT). Patrick et al. reported that several sepsis biomarkers may be affected by convective removal during renal replacement therapy, raising concerns about their reliability in patients undergoing CRRT. HBP was detected in effluent samples, but plasma levels did not show a consistent decrease [67,68]. These findings suggest that HBP may be relatively stable during CRRT; however, further studies using different hemofilters and adsorption profiles are needed before HBP can be considered reliably interpretable during CRRT. This issue is particularly relevant in transplant recipients with severe infection, delayed graft function, or postoperative renal support.

Beyond infection diagnosis, HBP has also been investigated as a marker of disease severity and prognosis in several non-transplant conditions. HBP has been reported to predict adverse events in chronic heart failure [69]. In critically ill patients with COVID-19, HBP levels increased with disease exacerbation and were associated with ventilation and perfusion abnormalities, sometimes preceding clinical deterioration [70]. In acute lung injury (ALI) and acute respiratory distress syndrome (ARDS), plasma HBP levels were higher than in cardiogenic pulmonary edema (CPE) and were associated with short-term mortality [71]. Han et al. further showed that serum HBP levels were higher in non-survivors than survivors and that adding HBP to the quick Sequential Organ Failure Assessment (qSOFA) score improved prediction of 30-day mortality [72]. Elevated plasma HBP levels at ICU admission have also been independently associated with early ICU mortality [73]. These findings are relevant to transplantation because early postoperative death is often related to infection, respiratory dysfunction, systemic inflammation, and ICU-level organ support. However, their application to transplant recipients requires dedicated validation.

Overall, HBP has potential as a candidate biomarker for post-transplant infection surveillance, inflammatory risk stratification, and graft injury monitoring, especially in settings characterized by neutrophil activation and endothelial dysfunction. The strongest transplant-specific evidence currently supports the use of BALF HBP for pulmonary infection assessment after lung transplantation. For kidney, liver, heart, and other solid organ transplants, the biomarker value of HBP remains largely informed by non-transplant evidence from sepsis, meningitis, cardiac surgery, COVID-19, ALI/ARDS, and ICU cohorts. Future transplant-specific studies should clarify the relationship between HBP levels and specific complications, including infection, DGF, PGD, rejection, and graft dysfunction. They should also define optimal sample types, sampling windows, diagnostic thresholds, and potential confounding factors, since HBP may also be elevated in non-septic shock, sterile inflammation, and other critical illness states [57] (Table 2).

## 5. Inflammatory Intervention Strategies and Potential Therapeutic Pathways for HBP

### 5.1. Direct Modulation of HBP Levels or HBP Activity

One potential approach is to reduce circulating HBP levels or neutralize its biological activity. Sterner et al. reported that heparin administration and CPB were associated with increased plasma HBP levels during cardiac surgery, whereas postoperative protamine administration reduced HBP levels [74]. These findings suggest that perioperative anticoagulation and heparin reversal may influence HBP dynamics. Heparin-based strategies have also been explored in severe infection. Kassem et al. reported that intravenous unfractionated heparin (UFH) was associated with reduced HBP levels and improved survival in patients with severe sepsis, with a more favorable effect than subcutaneous administration [75]. These findings suggest that UFH may modulate HBP-related inflammatory responses in selected septic patients. Nevertheless, the optimal use of UFH in critically ill patients remains challenging, as anticoagulation quality, activated partial thromboplastin time (APTT) monitoring, bleeding risk, and time to therapeutic range may all influence clinical application [76]. Another direct strategy is to block HBP binding to endothelial or immune cell targets. Neutralizing antibodies against HBP may theoretically inhibit HBP-mediated endothelial permeability and inflammatory amplification. In a mouse model of acute lung injury, inhibition of HBP-related signaling reduced pulmonary edema, inflammatory infiltration, and endothelial barrier disruption [18]. In addition, heparin derivatives may antagonize HBP by competitively binding to HBP and reducing its interaction with endothelial or inflammatory pathways. Previous studies have shown that such compounds can inhibit HBP-mediated bradykinin release and attenuate vascular leakage and inflammatory responses [22,77].

### 5.2. Indirect Modulation of HBP-Related Signaling Pathways

In addition to direct neutralization, several downstream pathways related to HBP activity may represent therapeutic targets. HBP has been shown to promote endothelial permeability and inflammatory activation through pathways involving TRIM21–p65, NF-κB signaling, glycolytic activation, and lactate-related metabolic regulation [18,27]. Silencing TRIM21 or applying ubiquitination inhibitors such as MG132 can attenuate HBP-induced endothelial injury in experimental models [18]. Similarly, inhibition of lactate transmembrane transport through monocarboxylate transporters (MCTs) can reduce NF-κB activation and TNF-α expression, supporting the role of lactate metabolism in inflammatory amplification [27,78]. Low-molecular-weight heparin (LMWH) has also been reported to reduce LPS-induced neutrophil retention and lung permeability, partly by inhibiting NF-κB nuclear translocation [27,78,79,80]. Other molecules, such as EDTA and fucoidan, have been reported to interfere with HBP binding to monocytes and reduce HBP-induced TNF-α production [81]. These findings suggest that blocking HBP-cell interactions may attenuate inflammatory activation. However, their potential use in transplant-related infection or rejection remains experimental and requires further validation.

### 5.3. Supportive Interventions That May Reduce HBP-Associated Endothelial Injury

Some supportive therapies may not directly target HBP but could reduce downstream injury associated with HBP-mediated vascular leakage and endothelial dysfunction. Albumin is one such example. Fisher et al. showed that albumin at concentrations of 20–30 g/L significantly reduced HBP- and thrombin-induced endothelial permeability, although albumin also induced IL-6 release from renal tubular cells, suggesting potential pro-inflammatory effects under certain conditions [82]. Clinical studies have also reported that albumin infusion may improve hemodynamics and reduce inflammatory mediators in selected patients [83]. In acute decompensated cirrhosis, HSA has been used to prevent or treat organ failure and may modulate immune cell responses and Toll-like receptor signaling without impairing phagocytosis, apoptotic cell clearance, or reactive oxygen species production [84]. Fluid strategy is also relevant to endothelial protection and graft perfusion. In critically ill adults, balanced crystalloids have been associated with fewer adverse kidney outcomes than saline in some studies [85]. In contrast, starch, dextran, albumin, and fresh frozen plasma have not consistently improved mortality compared with crystalloids, and starch may increase the need for blood transfusion and renal replacement therapy [86]. These data do not directly demonstrate HBP modulation, but they highlight the importance of fluid and endothelial support in inflammatory states characterized by vascular leakage.

In transplantation, endothelial protection is also relevant to chronic graft vascular injury. Cardiac allograft vasculopathy (CAV) is characterized by endothelial injury, diffuse intimal thickening, vascular inflammation, smooth muscle cell proliferation, and impaired graft perfusion. Although HBP has not been directly established as a mediator of CAV, HBP-related endothelial dysfunction may provide a biological rationale for further investigation. Vasoactive and vascular-protective peptides, such as apelin, have shown protective effects in experimental CAV-related injury models [43]. These approaches should be considered vascular-protective strategies with potential relevance to HBP-associated endothelial injury, rather than direct anti-HBP therapies.

### 5.4. Immunomodulatory Interventions with Theoretical Relevance to HBP

Perioperative glucocorticoids are widely used in transplantation and may indirectly influence HBP-related inflammation by reducing neutrophil activation, inflammatory cell adhesion, cytokine production, and lysosomal enzyme release [87]. These effects may contribute to the attenuation of ischemia–reperfusion injury and early inflammatory responses. However, the extent to which glucocorticoids directly regulate HBP release in transplant recipients has not been clearly established.

Dendritic cell (DC) maturation is another process relevant to graft immune outcomes. Immature DCs tend to promote immune tolerance, whereas mature DCs enhance antigen presentation and rejection-related immune activation. HBP may indirectly contribute to DC maturation by enhancing inflammatory mediator release and activation of antigen-presenting cells, thereby participating in the inflammatory environment that supports rejection. Clinical studies have shown that high-dose glucocorticoids can suppress pro-inflammatory DC activity and reduce DC maturation [88]. Therefore, HBP-related inflammatory signaling may be linked to DC maturation and rejection-associated immune activation, but the proposed HBP–DC axis remains hypothetical and requires experimental confirmation in transplantation models.

### 5.5. Future Directions for HBP-Targeted Therapy in Transplantation

Future therapeutic development may focus on more specific HBP-targeted strategies. HBP has been reported to regulate endothelial activation markers such as VCAM-1 and chemokines such as MCP-1, suggesting that it may serve as a pharmacological intervention point in endothelial inflammation [16]. Specific HBP inhibitors, neutralizing antibodies, or optimized heparin-derived compounds may be developed to reduce HBP-mediated vascular leakage, leukocyte recruitment, and inflammatory amplification. These therapies could theoretically be combined with conventional immunosuppressive regimens in transplant recipients with high inflammatory burden, severe infection, ischemia–reperfusion injury, or early graft dysfunction.

Overall, HBP-related intervention strategies include reducing HBP release, neutralizing HBP activity, blocking HBP-cell interactions, inhibiting downstream signaling pathways such as TRIM21–p65 and NF-κB, and alleviating endothelial barrier injury. At present, direct clinical trials targeting HBP in organ transplantation are lacking. Therefore, therapeutic discussion should distinguish direct HBP-targeted approaches from indirect anti-inflammatory and supportive interventions. Further studies are needed to determine whether HBP-targeted therapy can safely reduce transplant-associated inflammation without impairing antimicrobial defense or increasing bleeding and immunosuppression-related risks (Table 3).

## 6. Limitations

Several limitations should be acknowledged in the current review. First, transplant-specific evidence regarding HBP remains limited. Most available studies have investigated HBP in sepsis, acute lung injury, burns, cardiothoracic surgery, meningitis, and other infectious or inflammatory conditions, whereas direct studies in solid organ transplantation are scarce. At present, the most relevant transplant-specific evidence mainly comes from lung transplantation studies evaluating HBP in bronchoalveolar lavage fluid during pulmonary infection. Therefore, the proposed roles of HBP in post-transplant infection, ischemia–reperfusion injury, acute rejection, antibody-mediated rejection, chronic rejection, graft vasculopathy, and graft dysfunction are largely based on biological plausibility and extrapolation from non-transplant inflammatory models.

Second, prospective transplant-specific cohort studies are lacking. Existing evidence is insufficient to determine whether HBP levels are independently associated with specific transplant complications, such as primary graft dysfunction, delayed graft function, rejection, or infection-related graft injury. In addition, the optimal sample type for HBP measurement remains uncertain. Potential samples include plasma, serum, urine, bronchoalveolar lavage fluid, graft drainage fluid, and graft tissue, but their relative diagnostic and prognostic value may differ according to organ type and clinical scenario.

Third, the timing of HBP measurement and clinically meaningful thresholds have not been established in transplant recipients. HBP may change dynamically during surgery, ischemia–reperfusion, infection, rejection, renal replacement therapy, and intensive care support. These factors may influence its interpretation and limit its immediate clinical application. Furthermore, validated cut-off values for diagnosis, risk stratification, prognosis prediction, or treatment decision-making are not yet available in transplantation. Future multicenter prospective studies with standardized sampling protocols, organ-specific analyses, and clearly defined clinical endpoints are needed to determine whether HBP can be integrated into transplant-specific biomarker panels or therapeutic decision frameworks.

## 7. Conclusions

HBP is an activation-dependent neutrophil-derived protein involved in innate immune activation, endothelial barrier regulation, leukocyte recruitment, and inflammatory amplification. These biological properties provide a strong rationale for exploring HBP as a promising candidate biomarker and mechanistic target in transplant-associated complications, including infection, ischemia–reperfusion injury, graft dysfunction, rejection-associated inflammation, and vascular injury.

Current evidence from lung transplantation, sepsis, acute lung injury, cardiothoracic surgery, and other inflammatory conditions suggests that HBP may be particularly relevant in clinical settings characterized by neutrophil activation and endothelial dysfunction. In transplantation, these findings support further investigation of HBP for infection surveillance, graft injury monitoring, inflammatory risk stratification, and potential therapeutic modulation.

Future prospective transplant-specific studies are needed to define the temporal dynamics, sample sources, diagnostic thresholds, and organ-specific clinical value of HBP. Such studies will help determine how HBP can be integrated into biomarker panels and therapeutic strategies to improve the precision management of transplant recipients.

## Figures and Tables

**Table 1:** HBP-mediated inflammatory pathways and their biological roles.

Classification	Major Channels	Function/Mechanism of Action
Enhance vascular permeability	PKC/Rho kinase → MLC phosphorylation	Endothelium contraction and enlargement of intercellular spaces [23]
	β2 integrin-PI3K	Increased endothelial permeability [16]
	TRIM21-p65 (K63 ubiquitination)	VE-cadherin degradation, activation of Glycolysis [18]
	Calcium-dependent skeletal rearrangement	Endothelial space formation and increased vascular permeability [30]
	Contact system—KKS—B2 receptor	Promotes bradykinin production and enhances vascular permeability [22]
Recruiting leukocytes and inflammation amplification [16]	CCR2/FPRL1 receptor binding	Induce monocyte migration
	PI3K/AKT → MCP-1 secretion	Indirectly promotes monocyte recruitment
	p38 MAPK/NF-κB	Release of pro-inflammatory factors to amplify the inflammatory response
Non-classical inflammatory mechanisms	Lactate–NF-κB pathway	Lactic acid accumulation → NF-κB activation → TNF-α, IL-1β expression [27]
	Fcγ receptors (CD32, CD64) are upregulated	Enhances macrophage phagocytosis [28]

**Table 2:** The known biomarker performance of HBP under different clinical occasions.

Clinical Setting	Performance of HBP as a Biomarker	Evidence Type	Category	References
Neonatal sepsis	Sensitive blood markers to assess disease occurrence and severity	Non-transplant clinical evidence	Early diagnosis and stratification	[56]
MIRCS after cardiac surgery	Significantly higher coronary sinus HBP levels than the control group	Non-transplant clinical evidence	Early diagnosis	[57]
Meningitis	The highest diagnostic accuracy in distinguishing bacterial from viral meningitis; superior to lactate and PCT in identifying hospital-acquired meningitis and ventriculitis, while helping to address diagnostic difficulties caused by empiric antibiotic therapy	Non-transplant clinical evidence	Distinguish between bacterial and viral infections; identifying hospital-acquired meningitis and ventriculitis	[11,58,59]
Infection after lung transplantation	Sensitive markers of lung infection in BALF, better than lysozyme and a variety of inflammatory cytokines, which solves the problem of long time and low sensitivity of traditional microbial culture	Transplant- specific evidence	Distinguish between infected and non-infected	[36]
Sepsis	HBP increased rapidly in the early stage of infection, and performed better than PCT, CRT and lactate levels in detecting sepsis in patients with systemic infection. The reaction period is long, less affected by sampling time, and has stronger time stability	Non-transplant clinical evidence	Early diagnosis; Rule out and confirm concurrent organ dysfunction; prediction	[7,57,61,62,65]
AOSD	Serum HBP may be a useful diagnostic biomarker for assessing disease activity in patients with AOSD and differentiating AOSD from sepsis, but its accuracy needs to be improved.	Non-transplant clinical evidence	Distinguish between AOSD and sepsis; Distinguish between active and inactive AOSD	[63]
CRRT	The level of HBP in plasma is not affected by convection, which is better than that of PCT and CRP.	Non-transplant clinical evidence	Prognosis	[67]
Chronic heart failure	Predict the occurrence of future adverse events	Non-transplant clinical evidence	Prediction	[69]
COVID-19	HBP was significantly elevated and trended consistent with disease progression. Elevated HBP can be up to 5 days earlier than clinical manifestations and is strongly related to the patient’s lung ventilation and perfusion status	Non-transplant clinical evidence	Early diagnosis and stratification	[70]
Acute pancreatitis	HBP level is significantly elevated; No significant correlation between HBP levels and disease severity or intravenous fluid requirement	Non-transplant clinical evidence	Diagnosis	[41]
ALI/ARDS	Strong prognostic markers of short-term mortality	Non-transplant clinical evidence	Prognosis	[71]
ICU	Elevated plasma HBP levels at admission were independently associated with early ICU mortality	Non-transplant clinical evidence	Death prediction	[73]

**Table 3:** HBP intervention ideas and their trials.

Intervention Methods	Effect	Clinical Trials	References
Protamine is used postoperatively	Even if some patients develop infection, HBP remains below the risk threshold for organ dysfunction	Clinical trial with 40 patients	[74]
UFH	Significantly reduced HBP levels and improved survival in patients with severe sepsis	Clinical trial with 40 patients	[75]
Heparin	Reduces pulmonary edema and inflammatory infiltrate	Mouse model of acute lung injury	[18]
Heparin conjugate pretreatment	By competitively binding to antagonize HBP, it weakens its affinity with receptors and inhibits pro-inflammatory signals. Blocks HBP-mediated bradykinin release, reducing vascular leakage and inflammatory responses	Mouse model of systemic inflammation	[22,77]
CHC	Reduces preservation damage and improves early function	Clinical trial with 16 patients	[34]
Layer-by-layer heparinization of therapeutic cells: HBP-HSA and HBP-PEG-lipid conjugates	Effectively forms an anti-inflammatory environment and may inhibit IBMIR	In vitro experiments at the cell-molecular level	[79]
LMWH	Reduce LPS-induced neutrophil retention and lung permeability; Alleviate HBP-mediated inflammatory response by inhibiting NF-κB nuclear translocation	Rat ALI model	[80]
EDTA and fucoidan	Blocking HBP from binding to monocytes and attenuating its TNF-α effect	In vitro experiments at the cell-molecular level	[81]
albumin	It significantly inhibited the increase in permeability caused by HBP and thrombin, but at the same time induced the release of IL-6 from renal tubular cells, suggesting that it also had a certain pro-inflammatory effect	Clinical trial with 500 patients	[82,83]
HSA	Prevention and treatment of multiple organ failure in acute decompensated cirrhosis; Internalized by immune cells and regulates TLR signaling; It does not weaken the defense functions such as phagocytosis, removal of apoptotic cells and ROS production	Clinical trials	[84]
Balance crystal fluid	Intravenous fluids with balanced crystalloid fluids are associated with a lower incidence of adverse outcomes after normal saline surgery	clinical trial with 15,802 patients; Clinical trial (data analysis) with 30,020 patients	[85,86]
Vasoactive peptides	(pro-angiogenic peptide) apelin, which has a protective effect in experimental CAV injury	Mouse heart transplant model	[43]
Glucocorticoids	Neutrophil HBP release may be indirectly reduced by stabilizing lysosomal membranes, inhibiting inflammatory cell adhesion and cytokine release, inhibiting pro-inflammatory DC activity, and reducing maturation	Acute liver transplantation rejection rat model in rats	[87,88]
Inhibition of lactate transmembrane transport/direct blockade of NF-κB activity	The pro-inflammatory effect amplified by HBP through metabolic pathways is significantly weakened, thereby reducing the inflammatory cascade	Mouse peritoneal macrophage model	[27,78]
Ubiquitinated modifications targeting the TRIM21–p65 signaling axis	Reversing HBP-induced endothelial barrier disruption and glycolytic activation provides a new intervention direction for reducing vascular leakage and tissue damage	Mouse lung injury model	[18]

## Data Availability

Not applicable.

## Abbreviations

The following abbreviations are used in this manuscript:

HBP	heparin-binding protein
AR	acute rejection
IRI	ischemia–reperfusion injury
PCT	procalcitonin
CRP	C-reactive protein
LPS	lipopolysaccharides
TGF-β-R2	transforming growth factor-β receptor type 2
KKS	kinin-releasing enzyme-kinin system
AKI	acute kidney injury
MPO	myeloperoxidase
TLR4	Toll-like receptor 4
PGD	primary graft dysfunction
DGF	delayed recovery of graft function
CPB	cardiopulmonary bypass
SIRS	systemic inflammatory response syndrome
IMPs	immunomodulatory nanoparticles
ABMR	antibody-mediated rejection
NK	natural killer
CAV	coronary artery vasospasm
ROS	reactive oxygen species
DAMPs	damage-related molecular patterns
HMGB1	high mobility group protein B1
PRR	pattern recognition receptors
MIRI	myocardial ischemia–reperfusion injury
IMH	intramyocardial hemorrhage
MP	machine perfusion
IPC	ischemic preconditioning
HMP	hypogenic mechanical perfusion
NMP	normothermic mechanical perfusion
HOPE	hypogenic oxygenation perfusion
MIRCS	myocardial injury-related cardiogenic shock
BALF	bronchoalveolar lavage fluid
AOSD	adult onset still disease
RRT	renal replacement therapy
ALI	acute lung injury
ARDS	acute respiratory distress syndrome
CPE	cardiogenic pulmonary edema
qSOFA	sequential organ failure score
UFH	unfractionated heparin
APTT	activated partial thromboplastin time
CHC	Corline heparin conjugate
IBMIR	immediate blood-mediated inflammatory response
HSA	human serum albumin
LMWH	low molecular weight heparin
MCT	monocarboxylate transporters
DC	dendritic cell
PKC	protein kinase C
ICAM-1	intercellular adhesion molecule 1
IL-6	interleukin-6
CCR2	CC chemokine receptor 2
FPRL1	Formyl peptide receptor-like 1
MCP-1	monocyte chemoattractant protein-1
